# Compact Embedded Wireless Sensor-Based Monitoring of Concrete Curing

**DOI:** 10.3390/s18030876

**Published:** 2018-03-15

**Authors:** Joaquín Cabezas, Trinidad Sánchez-Rodríguez, Juan Antonio Gómez-Galán, Héctor Cifuentes, Ramón González Carvajal

**Affiliations:** 1 Department of Electronic Engineering, University of Seville, Camino de los Descubrimientos, s/n, E-41092 Seville, Spain; joaquin.cabezas@adevice.es (J.C.); carvajal@us.es (R.G.C.); 2 Department of Electronic Engineering, Computers and Automatic, University of Huelva, Ctra Huelva-La Rábida, s/n, 21819 Huelva, Spain; jgalan@diesia.uhu.es; 3 Department of Continuum Mechanics and Structural Analysis, University of Seville, Camino de los Descubrimientos, s/n, E-41092 Seville, Spain; bulte@us.es

**Keywords:** embedded sensor, concrete curing, monitoring, wireless networks, IEEE 802.15.4

## Abstract

This work presents the design, construction and testing of a new embedded sensor system for monitoring concrete curing. A specific mote has been implemented to withstand the aggressive environment without affecting the measured variables. The system also includes a real-time monitoring application operating from a remote computer placed in a central location. The testing was done in two phases: the first in the laboratory, to validate the functional requirements of the developed devices; and the second on civil works to evaluate the functional features of the devices, such as range, robustness and flexibility. The devices were successfully implemented resulting in a low cost, highly reliable, compact and non-destructive solution.

## 1. Introduction

Monitoring of concrete properties introduces many challenges in the design of non-destructive sensing systems. The miniaturization of the sensors taking advantage of technological advances is crucial for their placement inside the concrete. In recent years the growing importance of the so-called structural health monitoring is demanding intelligent systems based on sensors embedded into concrete, capable of controlling and monitoring the curing process mainly by measuring the temperature, or both temperature and humidity [1,2]. Other traditional approaches for structural health monitoring are based on accelerometers, strain gauges and displacement transducers [3,4,5]. However, the amount of works related to sensors embedded into concrete is limited by the obvious unfavorable conditions under which they have to operate.

Digital temperature and/or humidity sensors are widely known and their evolution continues to progress. These small systems incorporate the sensor, conditioning electronics, analog to digital conversion and digital processing necessary for connecting to a digital bus in one integrated circuit of reduced dimensions. Digital sensors provide advantages in terms of size, cost, reduced wiring, high noise immunity and direct connection to digital buses, allowing for the connection of large number of sensors without auxiliary electronics. However, the available digital temperature and/or moisture sensors cannot be used directly into the concrete, since their packages are not able to withstand the adverse conditions, especially in terms of sealing and compression. 

On the other hand, wired sensing increases the cost of the monitoring system [6]. Moreover, the added complexity would not make such a system feasible to be used for monitoring purposes on a construction site. A wireless approach would help to enable its use for on-site applications [7,8,9,10,11].

This paper describes our experience with the introduction and development of an experimental sensor embedded into concrete. The sensor exhibits temperature measurement capability. According to most of the available thermo-chemical models for concrete [12,13,14,15], by monitoring the evolution of the internal temperature of the concrete bulk, the effect of other factors, such as humidity, water to cement ratio, composition of cement, etc., on concrete curing are also considered. A specific package was designed to protect the sensor from the aggressive environment within the concrete. This sensor also incorporates significant additional features in terms of connectivity, measuring, remote programming and operation. To avoid affecting the concrete structure the embedded sensor occupies as small a space as possible. Thus, the sensor only comprises very few and small components. 

Concerning the integration of a wireless embedded sensor into an instrumentation system (generally into any measuring device), issues such as ease of connection or signal degradation due to the transmission process must be taken into account. The proposed sensor presents a number of features that are particularly useful for this application and that can be extended for structural health: (1) the energy consumption of the sensor node is optimized in such way that it can be powered by batteries. In this way, the energy supply is ensured throughout the lifetime of the node; (2) the simplicity of the proposed solution results in a low cost device, further guaranteeing robustness, accuracy and high resolution; (3) it can be connected directly to a standard instrumentation system. Thus, the developed embedded sensor includes an I2C full-duplex serial digital port (i.e., directly-transmitted and received signals are digital); (4) the sensor is equipped with a specifically designed virtual instrument, which allows acquiring and storing measurements in different formats, configuring the parameters of the sensor, reprogramming and controlling the sensor via Internet from a PC; (5) connection features in the developed sensor are significant, both in terms of quality (ease, robustness, immunity to noise, etc.) and the cost-savings involved in not having to transmit and condition analog signals outside the device; (6) a low cost and low power consumption solution with optimized size has been developed. 

The paper is organized as follows: Section 2 describes the main properties of the wireless sensor network in terms of type of standard, operation frequency, network topology, and transmission power. Section 3 deals with the hardware and software of the devices developed for the implementation of the system. Section 4 is devoted to experimental calibration procedures, testing and measurement. Finally, some conclusions are drawn in Section 5.

## 2. Considerations on Used Wireless Communication

Advances in low power wireless technologies made possible the immersion of a wireless node completely surrounded by the building material. Several wireless technologies, such as Bluetooth Low Energy, IEEE 802.15.4, ZigBee, Ultra-Wide Band (UWB) and Radio Frequency IDentification (RFID) [16,17,18] could be used to implement a wireless sensor network (WSN) within concrete, overcoming the problems related to the propagation of the electromagnetic waves through the structure and the node autonomy. 

Wireless sensor networks are composed of smart low rate devices or motes, which are generally battery-powered. The number of nodes can vary from a few to thousands. These networks allow for a dynamic network configuration, which is a feature particularly important for concrete curing, since a failure in one node is compensated by rerouting through other nodes. 

IEEE 802.15.4 was chosen for communications because in the last few years it has become a reference for wireless applications with low data rates and high energy efficiency. This standard, specifically designed for LR-WPAN networks, is a low-complexity protocol that offers low cost and energy features, as well as high versatility for the development of different topologies. 

### Network Topology

The network consists of a number of wireless nodes embedded into concrete, and needs to merge particular and commercial solutions. Regarding the radio propagation, the high signal attenuation and the antenna detuning must be considered. These issues can result in very high error rates, so the information exchange mechanism must adapt to it. In addition, the optimization of the energy consumption is degraded since more errors in the data transmission will require more retransmissions, and thus higher energy consumption. Finally, the transmission power must be high to avoid problems of attenuation. 

**Figure 1:**
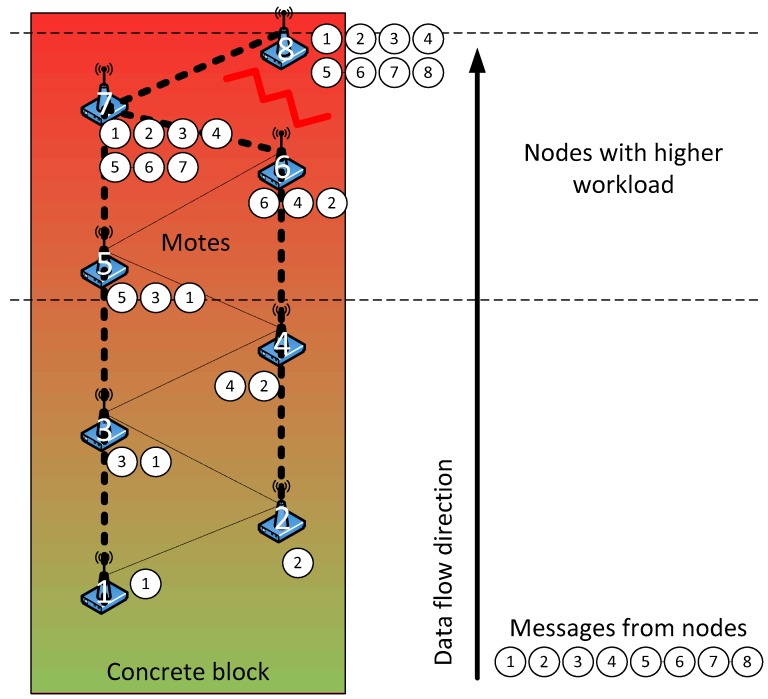
Network topology and associated problems.

It is common practice to place steel reinforcement to provide strength against tensile stresses and avoid excessive cracking of concrete. Moreover, the wet concrete is shaped in a mould comprised of formwork, which is most commonly steel or wood, being more problematic the presence of steel for the signal transmission. Therefore, if we want to monitor the concrete curing since when it is first poured, a careful design of the wireless network topology must be accomplished since the presence of steel may degrade the transmission of data. This fact imposes a wireless network topology of linear type, which involves an asymmetry in the network. Thus, the nodes that are further away have to send data through a long list of intermediate nodes. In turn, these intermediate nodes will waste part of their energy in the tasks of rerouting data. Figure 1 illustrates the network topology and the problems related to the data traffic. 

Considering the former problems, routing and synchronization algorithms have been designed to minimize the following characteristics: energy consumption, error rate, complexity and the probability of loss of a node. The energy consumption is decreased by synchronizing messages, whereas the rest of the time the node is idle. The use of very short messages allows balancing the workload between nodes, improving the error rate. Moreover, when a node performs a data transmission to all nodes within its coverage range (broadcast transmission), reliability is quite high since several nodes will receive the data message, so the loss of information is rather unlikely. The transmitting node waits for an acknowledgment message that validates its transmission. Then, it enters in low power state, reducing the time it is active. Concerning the complexity, the network is previously configured so that each node is assigned a task at the time when the network is created. The initial synchronization of the clocks is carried out in an outstation. Finally, to decrease the probability of losing a node an external watchdog is used, and different strategies will be applied depending on the level of the battery. 

## 3. Embedded Sensor Description

The smart wireless devices or motes basically consist of a radio module including the antenna, a processor, a power supply and sensors mounted on the mote itself or connected to it. The radio module transmits and receives data through the IEEE 802.15.4 communication standard. The processor controls all the functions of the node. The power supply consists of a battery that uses a high performance power converter. The motes buried in concrete include sensors which measure temperature. In addition, an external environmental mote has also been implemented to measure the ambient temperature, since the study of concrete curing requires the analysis of both internal and external variables. Some of the nodes in the wireless sensor network may communicate with a computer through a gateway. Taking into account the environment in the concrete, the requirements that have been considered for the design of these devices are the following: (1) low cost and low power consumption; (2) use of long-lasting energy sources and reduced size; (3) a robust radio technology; (4) use of interfaces to connect different sensor nodes; (5) a package suitable to protect the wireless node from the concrete but that does not affect the measure of temperature.

### 3.1. Developed Sensor Node

**Figure 2:**
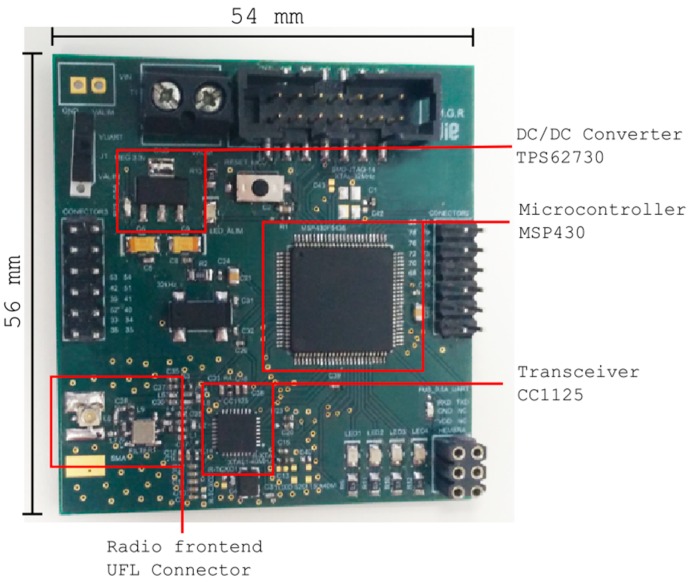
Wireless node (equipped with TI CC1125).

The mote consists of a microcontroller, a radio transceiver, a lithium-thionyl dichloride primary unit cell, an embedded antenna and a waterproof enclosure. The requirements of the microcontroller were ultra-low power consumption and low voltage. Two candidates were evaluated: a CC430F6137 (Texas Instruments, Dallas, TX, USA) which includes a CC1101 transceiver that allows several frequency bands below 1 GHz (868 MHz and 433 MHz), and a CC1125 transceiver, also from Texas Instruments, alongside with an MSP430 microcontroller (Texas Instruments, Dallas, TX, USA). The power supply consists of a 3.6 V battery connected to a TPS62730 DC/DC converter (Texas Instruments, Dallas, TX, USA) of 2.1 V and efficiency of 95%, especially suitable for wireless applications. The mote equipped with a CC1125 transceiver, shown in Figure 2, benefits from the excellent receiver sensitivity at low rate modes, reaching −129 dBm at 300 bps, and therefore was selected for the deployment. The dimensions of the PCB are 56 × 54 mm. 

The power consumption of each mote has been estimated considering the period of time at which measurements are taken as required by the concrete curing monitoring. The battery used was a LS14250 (SAFT, Levallois-Perret, France), with 1100 mAh capacity, a hermeticity guaranteed up to 110 degrees Celsius, and a small form factor (AA type, 50 mm length). This battery lasts around three months, which is more than enough time for the concrete curing monitoring.

**Figure 3:**
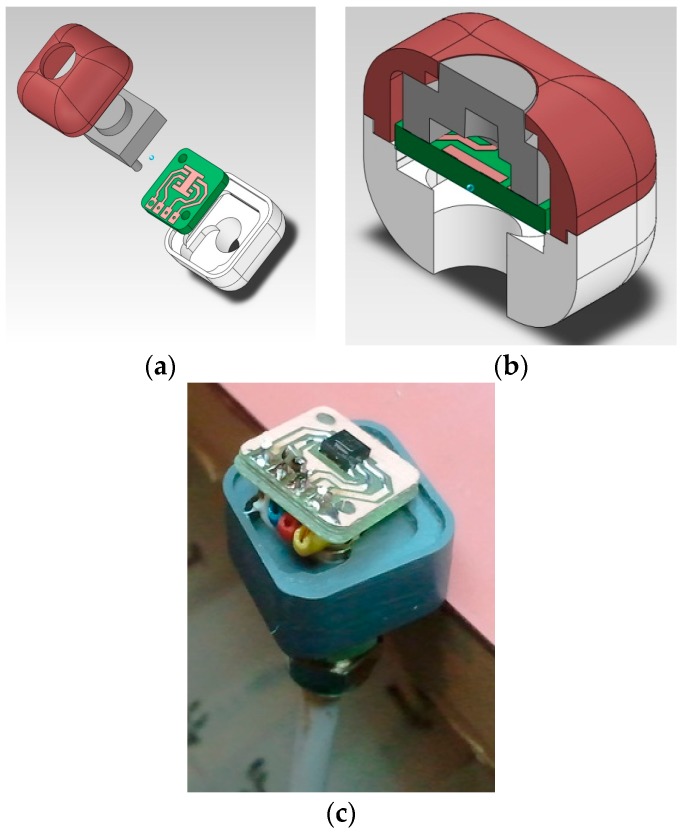
Embedded sensor (**a**,**b**) Detail of the construction (**c**) Real implementation.

Regarding the sensor, a specific package assembly was developed initially, with details of the construction shown in Figure 3. Concrete is an extremely aggressive material during early stages, reaching a pH of up to 13. As a protective element for the sensor, and at the same time acting as a temperature and moisture diffuser, an acetal cover has been designed and manufactured. Acetal has been used because it is a thermoplastic with characteristics superior to all other options, with an extraordinary impact resistance (high resistance to deformation), and is able to regain its elasticity. It is also very resistant to abrasion and traction. It is a perfect dielectric, so it is recommended for use in electrical and telecommunications applications, and it does not absorb moisture. The package must be designed in such a way as to not affect the measurement and transmission of temperature and moisture, while also protecting the electronics. A polypropylene membrane has been included in the package, aimed to allow separating the sensor from direct contact with the concrete while allowing the sensor layer to be exposed to the concrete environment to measure the parameters of interest. This is the key for a successful functionality.

The sensor layer consists of a commercial SHT21P humidity and temperature sensor (Sensirion, Staefa, Switzerland). This is a digital sensor that can be easily integrated into any circuit due to its ability to be directly connected to any microprocessor system. The claimed performance of the sensor shows good accuracy, good repeatability and good response time. Besides the capacitive relative humidity sensor and a band gap temperature sensor, the sensor chip includes an amplifier, an A/D converter, OTP memory and a processing unit. The developed sensor generates an electrical signal directly related to the temperature and moisture, which is subsequently converted inside the same device into digital format. This sensor transmits data in two packets, the first for the humidity reading and the second for the temperature reading.

A second alternative for the sensor was considered. The classical method of monitoring concrete curing is to only monitor the temperature profile. The DS18B20 digital temperature sensor from Maxim Integrated (San Jose, CA, USA) was chosen. This sensor is fairly precise, can provide up to 12 bits of resolution and works with any microcontroller using a single digital pin. The sensor covers the range of temperatures for the target application. Moreover, in order to avoid developing a specific package for the sensor, it was coated with a special lacquer and resin. The suitability of this protection was evaluated and tested to ensure accurate measurement of the temperature, but also protection from direct contact. This ensures a simpler solution which is crucial for developing a low cost and compact non-destructive sensing system suitable to be embedded into concrete.

Regarding the antenna, an internal antenna was always used, because of the risks related to maintaining the watertightness, and also impact risks with the aggregates of concrete (inert granular materials such as sand, gravel or crushed stone) being poured from the mixer truck to the moulds to be filled. Different antenna types were used, these being a dipole quarter wave (85 mm), a custom design on a PCB and a chip antenna. Our final design uses a dipole quarter wave antenna with an RP-SMA connector. 

### 3.2. Software

The language used in programming is almost entirely a C variant for microcontrollers which intended to be as portable as possible, so excluding lower level parts (drivers and statement types, memory or basis functions) it can be read as ANSI C. In any case, this microcontroller comes with very limited memory, so it is often preferred to include assembly code directly, although this is usually limited to certain functions related to wireless transmission. The firmware of the microcontroller is programmed by Code Composer or IAR for a device from the MSP430 family. 

The MSP FET430 programmer-debugger connects to the device via the JTAG or SWD protocol and allows for the step by step debugging and the use of breakpoints. Concerning the wireless controller, it is programmed using the protocol stack from Texas Instruments (SimpliciTI). It has been used for the programming of the nodes in different phases, and for the implementation of network algorithms. Synchronization processes and waiting periods have had to be defined as the protocol stack does not implement them. 

**Figure 4:**
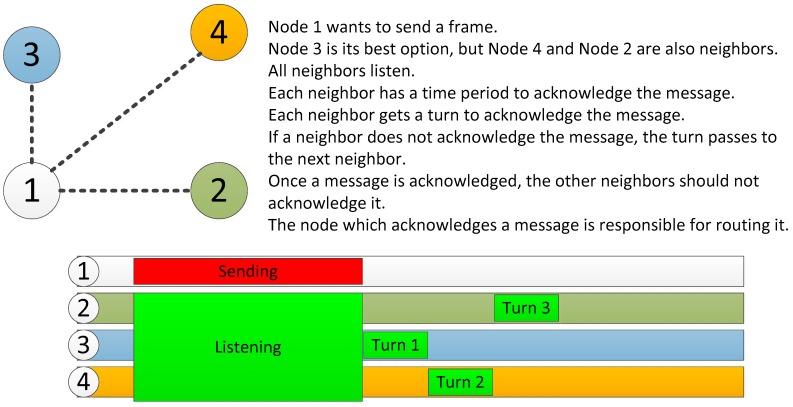
Simplified routing mechanism.

Figure 4 shows the simplified routing mechanism. First, a static routing has been chosen for power saving, but also considering the possibility of errors within the nodes. Thus, if the node 3 has low battery, it may decide not to acknowledge the message from node 1 to avoid having to route it, leaving this task to other nodes. These nodes could also reject it; then node 1 would detect that none of the nodes has accepted its message and would wait for the next turn, but this time it would include a field of alert, informing its neighbors that they must transmit the message in turns in order to spread the payload. 

**Figure 5:**
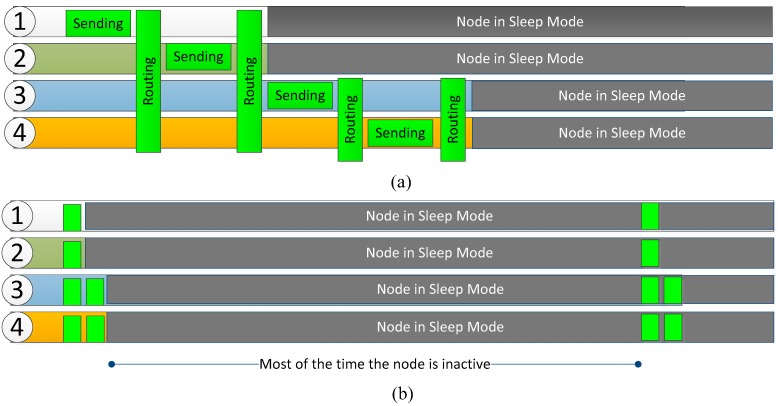
(**a**) Data sending spread out in intervals; (**b**) Power saving mechanism.

Sending messages will always be coordinated in stages as shown in Figure 5a, with an upstream direction according to their numbers as shown in Figure 1. As this network is only used for monitoring, it is only necessary to move upwards. This allows nodes with low identification number to enter the idle state first, providing a balance of energy consumption, since these nodes will be the first ones sending data (they are placed in the lower part of the concrete structure). 

The time required to send data always corresponds to a very low percentage of the total, so that the duty cycle will be below 1%, which allows for the device to be inactive most of the time (deep sleep state) as shown in Figure 5b; only the clock circuitry is enabled in order to know when the devices must wake up again. 

Thanks to these mechanisms a very low energy consumption is achieved and the use of small batteries becomes appropriate.

### 3.3. Monitoring and Control System

The monitoring application is a program that receives and stores data from the sensor nodes, and shows the data read from the sensors in a graphical user interface. The software has been developed with additional features in order to provide flexibility. Thus, the user can identify each node. The data from the sensors is periodically sent and stored within the database, and it is visualized in real time. In addition, the sampling period can be modified by the user. In fact, during the first days after the concrete is poured, the activity of the curing process is greater, requiring a large number of measurements. Some alarms received from the sensors, related to battery failure and voltage values lower than a preset threshold, have also been included. 

**Figure 6:**
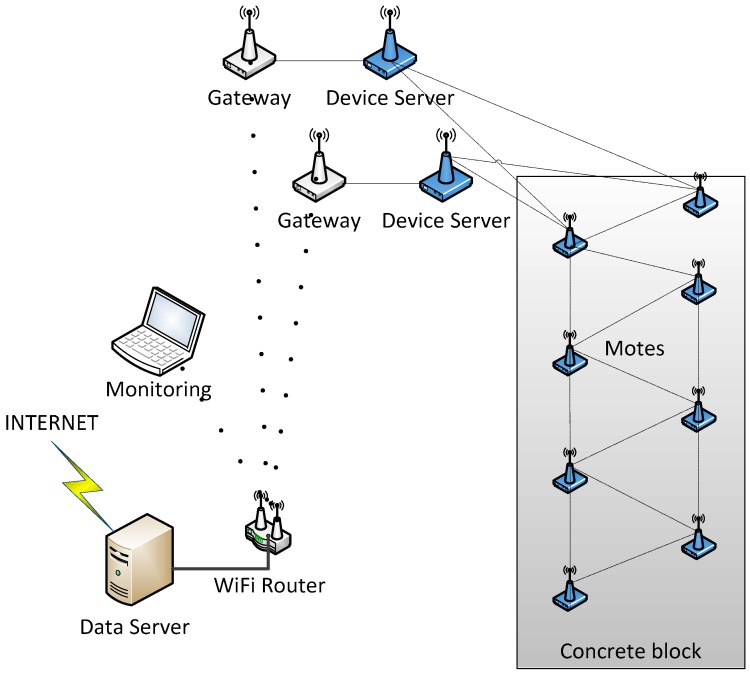
Scheme of the monitoring system based on the wireless sensor network.

Figure 6 shows the infrastructure required for the monitoring system. It consists of the motes inside the concrete, a gateway (NPort W2150A, Moxa, New Taipei City, Taiwan) between the measurement network and the data network, a WiFi router (AWK-4121, also from Moxa), a monitoring device and a storage device.

## 4. Experimental Results

Traditionally, for conventional Portland concrete a limit of 3 days for curing, 7 days for rapid hardening, and 28 days for the development of the characteristic strength of the concrete is considered. Considering a tradeoff between the accuracy of a thermo-chemical-mechanical model that could analyze the concrete curing and the use of a wireless sensor network with motes supplied from batteries, the sampling period of temperature for the sensors was set as follows: four measurements every hour for the first three days. From day four, one measurement every hour until the seventh day. Between day 7 and day 28, one temperature measurement is performed every three hours. To save power consumption, several sensor readings are stored and sent together in the same message. This approach provides lower power consumption in the radio module, but the longer frames increase the error rate. Thus, a maximum of 128 bytes per frame was set. The estimation of power consumption, including a safety margin, determines a minimum battery capacity of 707 mAh. The average power consumption of each mote has been estimated using a Simulink model and considering each component separately. After the model, real world estimations have been taken into account, like battery discharge caused by temperature, loss of efficiency on components and non-linear effects such as communication issues. Therefore, the selected battery has a capacity of 1100 mAh. 

### 4.1. Calibration and Testing

**Figure 7:**
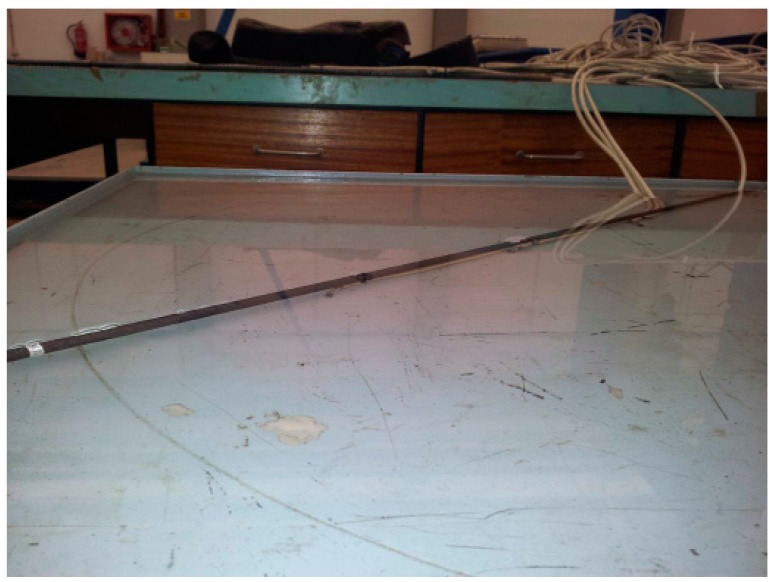
Sealing testing with sensors immersed in water.

Once the DS18B20 sensor was chosen all efforts were devoted to checking the suitability of the protection based on lacquer and resin. The accuracy and linearity of the sensor were monitored, as well as the effect that the protection had on the accuracy. Nevertheless, the suitability of the package in Figure 3 for the SHT21P sensor was also determined testing the ability of the acetal material and the poly propylene membrane to protect the sensor and also its effect on the measurement accuracy. This fact will allow evaluating the acetal material to be used to protect the wireless board in Figure 2. First, the effect of the lacquer and resin on the sensor response was tested within a water container, since it allows simultaneously checking the sealing of the sensors and the accuracy, as shown in Figure 7.

**Figure 8:**
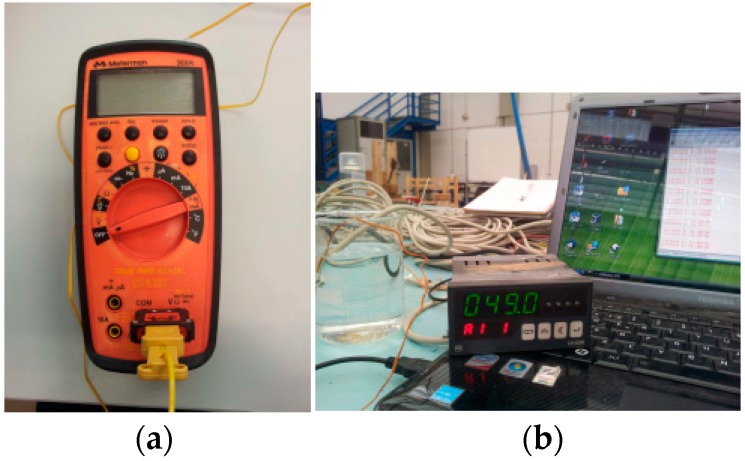
Thermocouples used for the sensor calibration. (**a**) TerEle, (**b**) TerEst.

**Figure 9:**
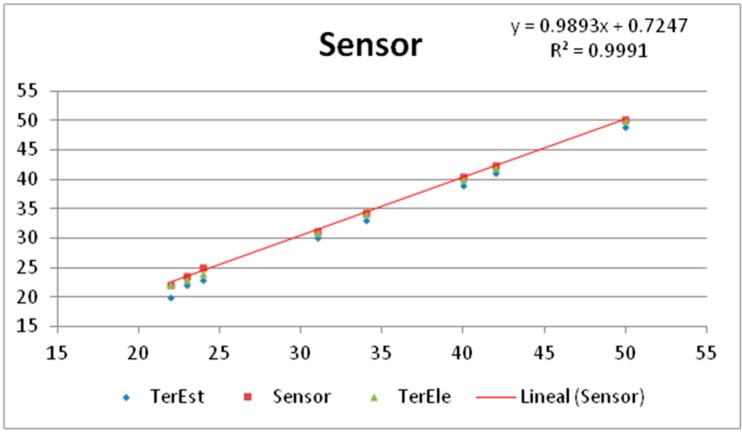
Calibration of a sensor with respect to the thermocouple references TerEst and TerEle.

Figure 8a,b show the thermocouples, named TerEle and TerEst respectively, used as reference for the calibration. The sensor was found to work accurately when compared to the temperature readings of the thermocouples with PT100 probes, as shown in Figure 9. Note that the sensor features a similar evolution to that of the references (0.99) with an offset of only 0.72 ± 0.5 °C. The results showed a proper operation without communication problems. 

**Figure 10:**
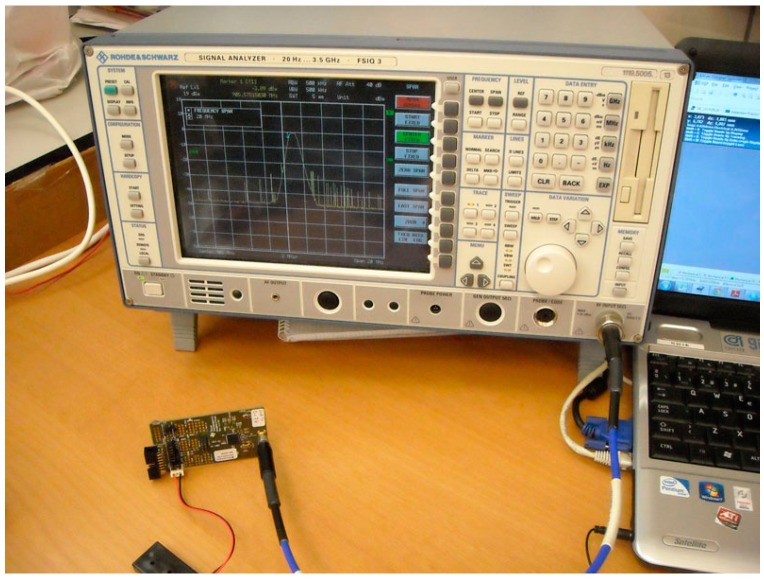
Test of output power level for the output signal of mote.

On the other hand, the transmission of each node was analyzed under different situations, especially considering the power of the output signal, which is a critical parameter taking into account the attenuation due to the concrete and the steel. Figure 10 shows the mote and a FSIQ 3 spectrum analyzer (Rhode & Schwarz, Munich, Germany). The tests were carried out both open air and inside concrete.

**Figure 11:**
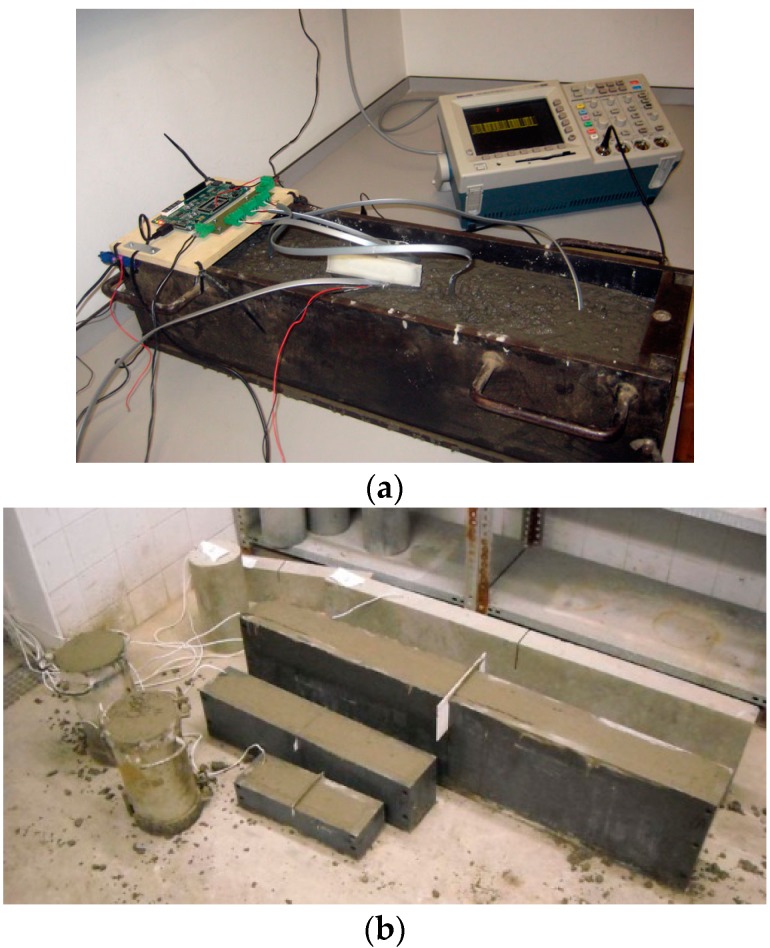
Preliminary test. (**a**) Data acquisition board to gather physical signals from several sensors (**b**) Mote buried in several specimens of concrete.

A preliminary test was performed to verify the validity of the specifications on several specimens of concrete as shown in Figure 11. Figure 11a also shows the data acquisition board used to gather physical signals from several sensors. The aim of this test is to determine if the sensors can perform inside a concrete specimen in order to replicate, as closely as possible, the construction site conditions. The specimens are created using a steel mould and filled with concrete. This test allows determining the effect of the combination of formwork and concrete on the transmission of data. In addition, the test also examines the transmission distance of the sensors. The radio transmission was tested by an emission test at fixed intervals of a data frame. As expected, the effect of concrete, rebar and formwork reduced the transmission distance of the system. When the moulds were taken out of the specimens, the transmission distance doubled. The design has been optimized to achieve a percentage of packets successfully received above 95%. The data was transmitted since the first pouring of concrete for 28 days. These results are obtained for a transmission distance of approximately two meters. Results confirmed the feasibility of the proposed approach. There were no changes in the transmission of data in the early stages of concrete pouring when it is wetter.

A simple LabVIEW program was designed to interact with the sensors, logging the time, temperature readings and also from which sensor the data was received. 

### 4.2. Construction Site

The designed motes and the wireless sensor network were introduced in a construction site. The place is located in a viaduct of the high-speed Antequera-Granada railway in the region of Andalucia, in the South of Spain. 

The preliminary study of the location of the motes determined the use of the 869.4 MHz to 869.65 MHz band given by ETSI EN 300 220 and short-range device (SRD)of unspecified use, with a transmission power of 500 mW (+27 dBm). This need arises from the distances to be treated being around three meters, which are larger than the ones tested in the laboratory, and therefore the dynamic range should be increased. Thus, to reach the mentioned power level, it is necessary to include in the design an output power amplifier and a linear noise amplifier to improve the reception of signals.

**Figure 12:**
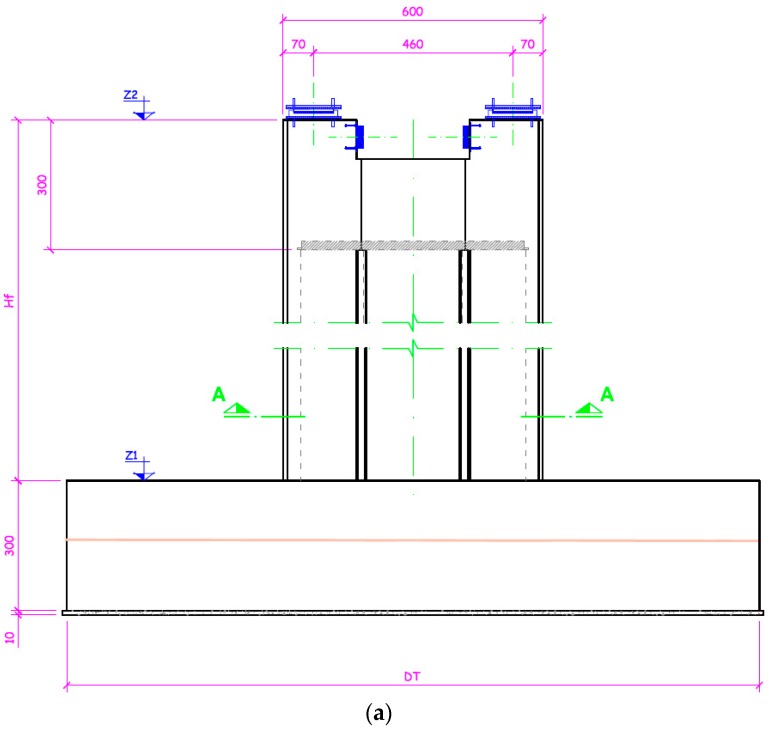

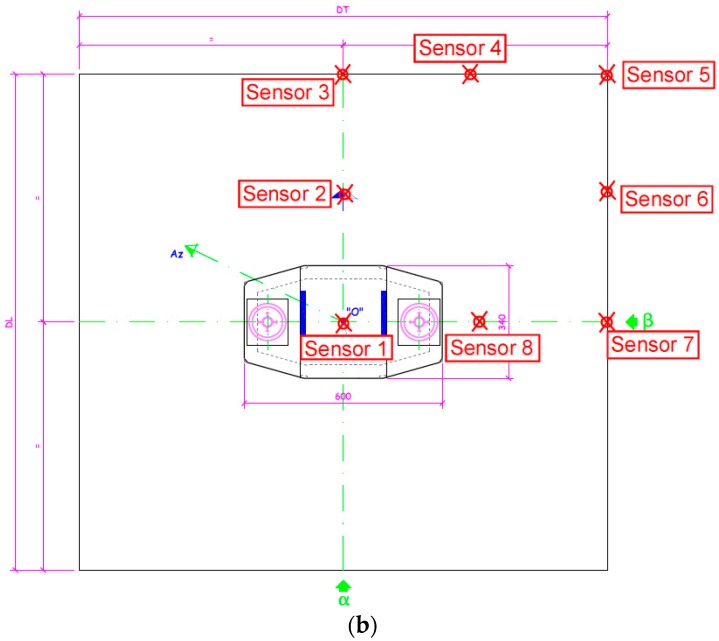
(**a**) Construction element and (**b**) location of sensors.

Figure 12 depicts the construction, the dimensions in centimeters and the location of each sensor. It consists of a footing to support a shaft. The dimensions of the footing are 8 × 11 × 3 m. The placement of the sensors is performed in the middle plane 1.5 m above the base. This location was carefully chosen from experimental results.

For a distance of 1.5 m the motes can transmit from within the concrete to the outside, with a signal loss between 48 dBm and 64 dBm. Given an initial signal of −36 dBm and an error margin, it’s the maximum distance where a sniffer can receive packets in order to debug the deployment, considering a sensibility of −102 dBm. Eight points of measurement are taken, as indicated in Figure 12b. The devices must be installed prior to pouring the concrete, and placed in preset positions using clamps or flanges to ensure that the measuring point corresponds to the one required. Each sensor is installed and tested individually by placing the antenna in different positions. Due to the nature of the test and the inability to repeat the process, several repeaters where included in the structure, to assure transmission of the values and to provide redundancy.

**Figure 13:**
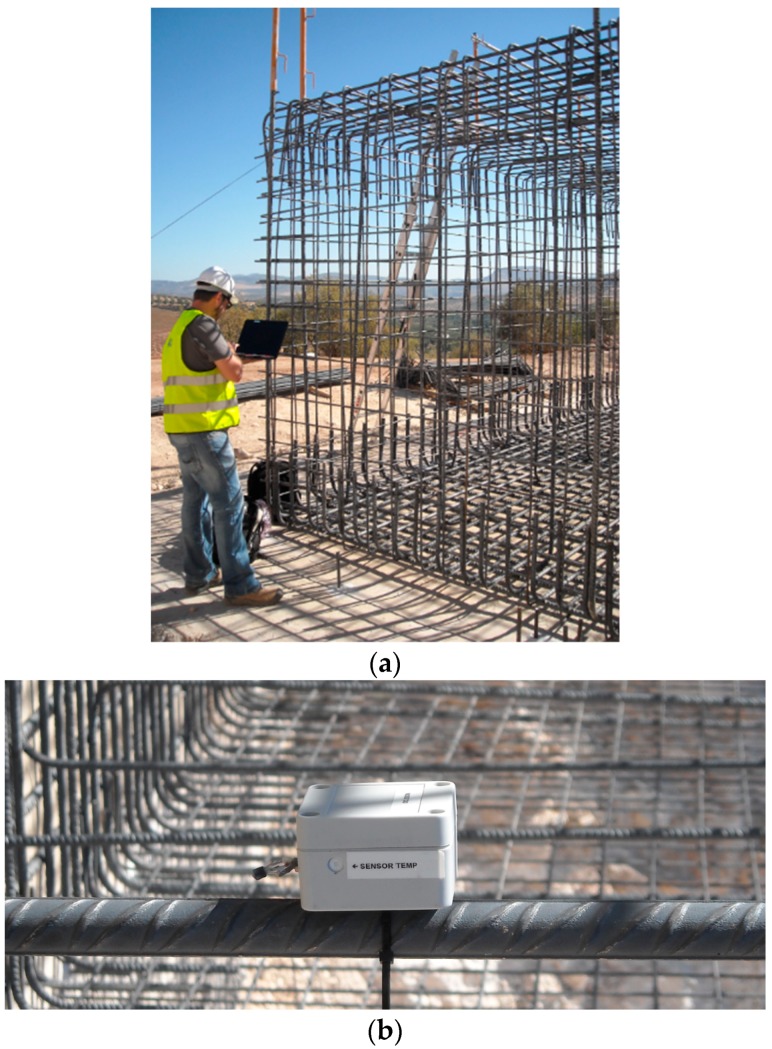
(**a**) Operator testing a sensor on the steel structure; (**b**) Detail of the mote tied to a steel bar.

Figure 13a shows an operator testing a mote on the structure, and Figure 13b shows a mote tied to a steel bar. For the wireless sensing node, a 4 mm thick acetal box has been used. The final dimensions of the prototype wireless node package are 85 mm width and length, and a height of 60 mm.

**Figure 14:**
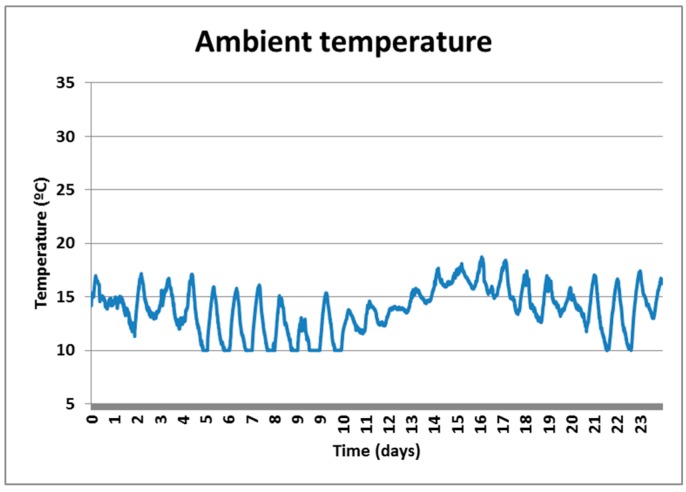
Ambient temperature profile.

Figure 14 shows the ambient temperature profile since the first hours that the concrete is poured up to day 28, respectively. Note that the variations in temperature can reach almost 10 °C.

**Figure 15:**
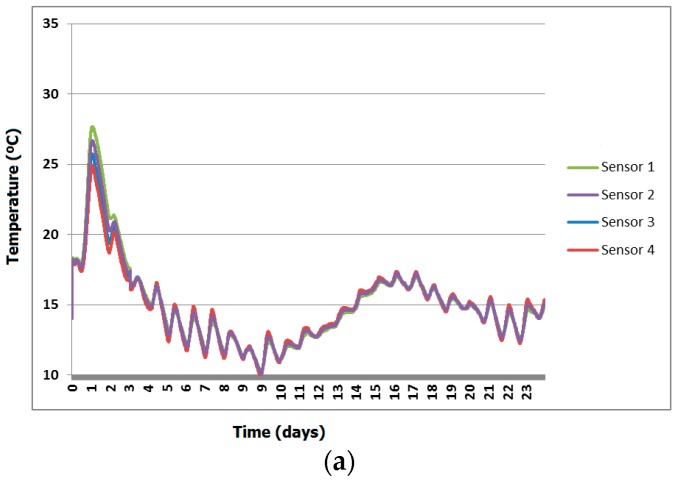

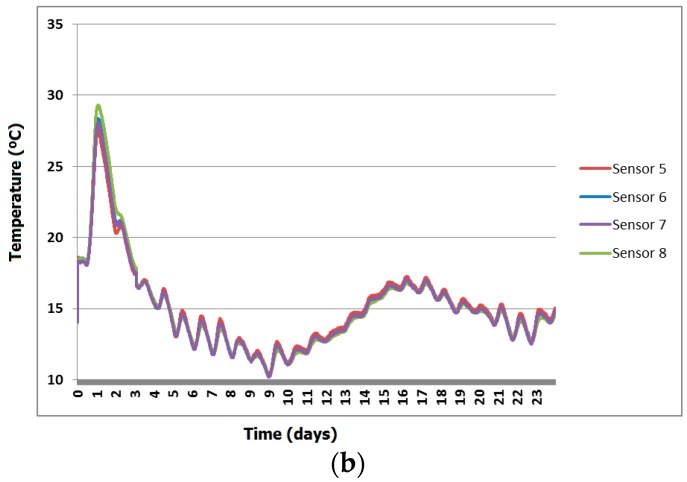
Temperature profile of concrete from sensors. (**a**) Sensors from 1 to 4. (**b**) Sensors from 5 to 8.

Figure 15 shows the temperature profile of the sensors located into the concrete. Note how the temperature of the concrete increased during the first hours due to the reaction between cement and water (hydration process). Once the temperature reaches a peak, then it starts to drop following the fluctuations of the ambient temperature. It can also be seen that the sensors 3 and 4 provide a slightly lower temperature, probably because they are located at the edges of the structure and the heat was dissipated away.

The higher temperatures inside the concrete structure did not affect the operation of the wireless sensor network, and measurements were gathered with the expected sensor accuracy, which is protected from impacts produced during the pouring process. 

The applicability of the developed monitoring system in civil engineering is evident. For example, by considering a thermo-mechanical model of the hydration kinetics of the cement (see for example [15]), the degree of hydration of the cement can be controlled by monitoring the evolution of the temperature with time. These experimental measurements provide useful information to validate the theoretical predictions and to check whether the hydration process inside the concrete bulk is developing correctly. Combining these measurements with those of the mechanical properties of concrete at early age and a convenient thermo-mechanical model of the structure (by using a finite elements model, for example) provides a powerful tool to determine the most convenient stripping time and, subsequently, the associated costs can be reduced. Table 1 shows the results of the main mechanical parameters, such as the compressive strength (*f_c_*), the Young’s modulus (*E*) and splitting tensile strength (*f_st_*) of the employed concrete at early age and at 28 days. These results were obtained in laboratory by means of standard tests on cubic and cylindrical concrete specimens [19].

**Figure 16:**
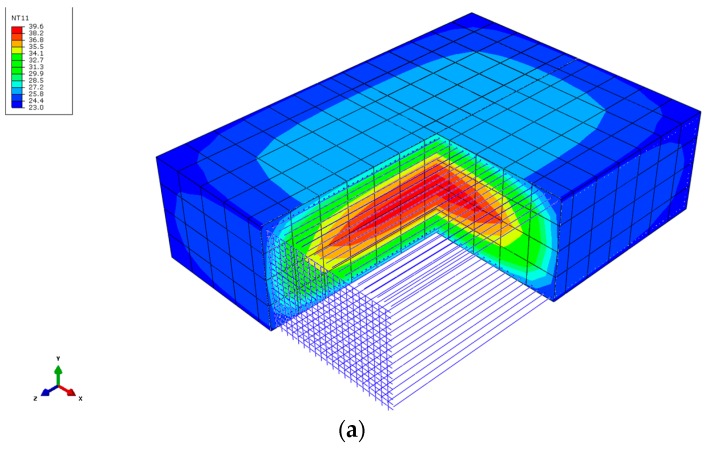

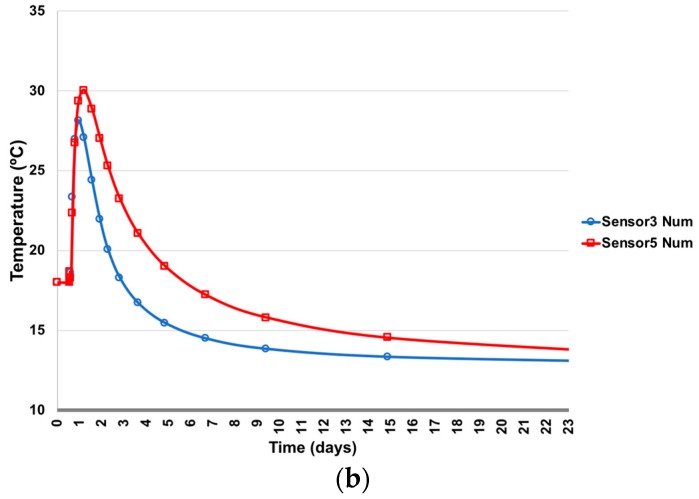
(**a**) Thermo-chemical-mechanical FEM model of the footing; (**b**) Numerical prediction of the evolution of temperature with age for sensors 3 and 5.

In order to confirm the reliability of the results, a thermo-chemical-mechanical finite element model (FEM) of the concrete footing has been implemented in ABAQUS. Details about the hydration kinetics modeling of the concrete employed in this analysis are given in [15]. The composition of the concrete in this work was 1/0.43/2.31/3.07/2.57/0.009 (cement/water/sand/gravel/superplasticizer) with a class II Portland cement content of 325 kg/m^3^. The concrete parameters necessary to perform the thermal analysis were a density of 2240 kg/m^3^, a coefficient of thermal expansion of 10^−5^ K^−1^, a thermal conductivity of 2.5 W/mK, a specific heat of 922 J/kgK, a film coefficient between concrete-air of 9 W/m^2^∙K and a room temperature of 18 °C. The behavior of the material was considered linear and elastic with a Young modulus ranging with age according to the values indicated in Table 1. Figure 16a shows the geometry and discretization employed in the FEM model for the concrete and the steel rebars, as well as a view of the internal profile of temperatures obtained for concrete at 7 days. In Figure 16b the evolution of the temperature with age for the nodal points of the FEM model corresponding with the location of sensors 3 and 5 (Figure 12b) are shown.

As observed from the results shown in Figure 16b, the evolution of the temperature with age predicted by the numerical model is in good agreement with that measured by the sensors placed in the structure. Obviously, the values of the numerical prediction are not exactly the same than those obtained experimentally because of several factors, which are difficult to consider in the model. For example, variation of the room temperature between day and night was not considered, the film coefficient was estimated from literature [19] and not experimentally obtained, and the temperature of the soil was also estimated, etc. However, the maximum values of the temperature given by the sensors, the age at which these values were obtained and the evolution of the concrete temperature after the peak value (cooling) are quite similar, which reflects the reliability of the experimental measurements.

## 5. Conclusions

A non-destructive wireless sensing system has been implemented, with a low cost and compact design suitable to be embedded into concrete for monitoring the curing process. This application demands small sized sensing nodes with low power requirements, complicating the design. Preliminary tests were accomplished in the laboratory to validate the accuracy and the protection of the developed sensors, and the data transmission to a data acquisition system located outside the concrete. Finally, the wireless system was used in a civil construction and the data from the sensors was successfully transmitted during the period required to monitor the concrete from the first pour.

## Figures and Tables

**Table 1:** Main mechanical parameters of the employed concrete at early ages and 28 days.

Age (Days)	*f_c_* (MPa)	*E* (GPa)	*f_st_* (MPa)
1	20.1	18.9	1.9
2	26.7	19.8	2.1
3	28.2	20.7	2.3
7	36.2	24.4	2.8
28	43.9	28.4	4.3
